# Gadolinium Nanoparticles: Emerging Platforms Beyond Imaging for Drug Delivery and Theranostics

**DOI:** 10.3390/pharmaceutics18030358

**Published:** 2026-03-13

**Authors:** Amir Nasrolahi Shirazi, Rajesh Vadlapatla, Ajoy Koomer, Heyam Zayed, Paris Marabut, Keykavous Parang

**Affiliations:** 1 Department of Pharmaceutical Sciences, College of Pharmacy, Marshall B. Ketchum University, 2575 Yorba Linda Blvd., Fullerton, CA 92831, USA; rvadlapatla@ketchum.edu (R.V.); akoomer@ketchum.edu (A.K.); heyamzayed.cop28@ketchum.edu (H.Z.); parismarabut.cop29@ketchum.edu (P.M.); 2 Center for Targeted Drug Delivery, Department of Biomedical and Pharmaceutical Sciences, Chapman University School of Pharmacy, Harry and Diane Rinker Health Science Campus, 9401 Jeronimo Rd., Irvine, CA 92618, USA; parang@chapman.edu

**Keywords:** gadolinium nanoparticles, drug delivery, metal nanoparticles

## Abstract

Gadolinium nanoparticles (GdNPs) have gained increasing attention as multifunctional metal-based nanoplatforms that extend far beyond their traditional use as magnetic resonance imaging (MRI) contrast agents. Their specific magnetic properties, tunable physicochemical features, and tunable biocompatibilities with biocompatible coatings give them great potential as drug delivery and theranostic applications. They offer greater stability, lower systemic toxicity, and more surface modification options compared to molecular gadolinium chelates. The functionalized GdNPs not only show excellent properties as drug carriers for their specific indications but also serve as agents in various imaging modalities with superior therapeutic efficacy by means of radio sensitization and magnetically assisted delivery. Note too that GdNP-based formulations have demonstrated synergistic activity when administered with chemotherapeutic agents such as doxorubicin. GdNPs have demonstrated promising preclinical outcomes, and their clinical translation remains restricted due to a number of scale-up constraints, long-term safety challenges, pharmacokinetics, and regulatory problems. This review provides information on the use of GdNPs, their key physicochemical and magnetic properties, ligand engineering for targeted delivery, and biological mechanisms of their theranostic performance.

## 1. Introduction

### 1.1. Inorganic Nanoparticles in Drug Delivery

Inorganic nanoparticles containing metals can be divided into three major categories, including metal nanoparticles, metal oxide nanoparticles, and quantum dots.

Metal nanoparticles (MNPs) are nanomaterials composed of a single elemental metal and can exist as individual atoms or clusters of many atoms [1,2]. Common examples include Au, Ag, Pt, Cu, Pd, Re, Zn, Ru, Co, Cd, Al, Ni, and Fe [3,4,5], and most metals can be synthesized into nanoparticles using either top-down or bottom-up approaches [6]. They are typically produced as colloidal suspensions or solid particles through fabrication methods such as bio-assisted synthesis, hydrothermal processing, and microwave-assisted techniques. These nanoparticles exhibit distinctive physicochemical properties including size, surface to volume ratio, pore size, electronic surface charge, structural characteristics (crystalline or amorphous), morphology (spherical or cylindrical), and sensitivity to external factors (e.g., humidity, temperature, light) along with exceptional functional features such as localized surface plasmon resonance (LSPR), high chemical reactivity, and broad electromagnetic absorption. Their excellent optical, optoelectronic, catalytic, and also antimicrobial/anticancer/antiviral properties have also contributed to their broad utility across diverse applications [7,8].

Metal oxide nanoparticles are produced by altering the properties of their parent metals. For example, iron-based nanoparticles (FeNPs) readily oxidize to iron oxide at room temperature in the presence of oxygen, resulting in increased reactivity compared with unoxidized FeNPs [9,10]. Most metal oxide NPs are synthesized through physical or chemical methods, as these materials can be reduced in size by mechanical forces (e.g., within a rotating reactor) or through their heightened chemical reactivity [11]. Other examples include aluminum oxide (Al_2_O_3_), iron oxide (Fe_2_O_3_), magnetite (Fe_3_O_4_), silicon dioxide (SiO_2_), and titanium dioxide (TiO_2_). These nanoparticles generally exhibit enhanced properties such as improved surface area, catalytic activity, tunable electronic, optimal properties, and biological interactions relative to the bulk metals from which they are derived [3,12].

Quantum dots are nanoscale semiconductor particles distinguished by their extremely small size and unique optical characteristics. Typically composed of materials such as cadmium selenide (CdSe), cadmium telluride (CdTe), or lead sulfide (PbS), they generally range from 2 to 10 nm in diameter. With tunable emission characteristics and high photostability, quantum dots are widely used in display technologies, solar cells, and bioimaging [13].

The review is devoted to MNPs, specifically to gadolinium nanoparticles (GdNPs), as emerging multifunctional nanoplatforms for biomedical applications. In the first section, we summarize the application of metal nanoparticles in drug delivery and compare key classes of systems with respect to their physicochemical properties, imaging capabilities, biocompatibility, and therapeutic potential such as AuNP, iron oxide, and GdNPs.

### 1.2. Metal Nanoparticles in Drug Delivery

MNPs have received much interest in nanomaterials research due to their size-dependent properties and extensive functional versatility. Thus, their adjustable size, high surface reactivity, and ability to incorporate multiple functions (e.g., simultaneous targeting and imaging), and controlled drug release, further give MNPs distinct advantages over other drug delivery platforms [1].

Widely studied MNPs include gold (AuNPs), silver (AgNPs), iron oxide (Fe_3_O_4_ NPs), platinum (Pt NPs), zinc oxide (ZnO NPs), selenium (SeNPs), and GdNPs. These nanomaterials can be produced by chemical, physical, or biological synthesis methods and have been widely studied due to their remarkable properties, enabling their use for various therapeutic, medical, and diagnostic purposes [1,6,8,14].

Fe_3_O_4_ NPs possess superparamagnetic properties, which allow them to be directed by external magnetic fields and to work as competent magnetic resonance imaging (MRI) contrast agents. When functionalized with polymers or peptides, these nanoparticles can be guided with high accuracy to tumors or inflamed tissues, thereby enhancing targeted drug delivery [15]. Additionally, their inherent biocompatibility and biodegradability support their integration into advanced theranostic platforms, where they enhance cellular internalization and the controlled release of therapeutics such as 5-fluorouracil (5-FU) [16].

Zinc oxide nanoparticles exhibit pH-responsive behavior which allows them to function differently in acidic environments generated by tumor tissues. This controlled process releases the molecular drug in a time-dependent manner and generates reactive oxygen species (ROS). This would make the nanoparticles cytotoxic against cancer cells [17].

Selenium nanoparticles (SeNPs) show strong antioxidant, anticancer, and immunomodulatory activities, inhibiting tumor growth [18] through apoptosis and redox regulation while exhibiting low toxicity toward normal cells [19]. Functionalization further enhances their targeting ability and therapeutic efficacy [20].

Gd-based materials have been extensively used in biomedical applications, particularly as MRI contrast agents in MRI, owing to the strong paramagnetic properties of Gd^3+^ ions [21,22]. Though various metal nanoparticle systems have shown promising properties in drug delivery and theranostics, GdNPs offer several distinctive advantages that motivate their continued investigation and development. Compared to other widely studied metal nanoparticles, GdNPs exhibit unique characteristics that position them as particularly attractive platforms for integrated diagnostic and therapeutic applications, including MRI contrast capabilities, unique theranostic properties, biocompatibility profiles, and multifunctional integration. Due to Gd’s pronounced paramagnetism, GdNPs, traditionally used as MRI contrast agents, have been advanced into sophisticated theranostic systems.

The multifunctional capabilities of GdNPs extend beyond imaging to encompass therapeutic modalities. Unlike AuNPs, which primarily rely on photothermal effects, or SeNPs, which function mainly through redox modulation, GdNPs can be engineered to integrate multiple therapeutic mechanisms within a single platform. Embedding imaging functionality into other modalities (e.g., targeted drug delivery, photothermal therapy) enables real-time visualization of disease sites and enables treatment. The ability of GdNPs to serve as targeting agents has been improved via different strategies. This combined diagnostic–therapeutic capability provides significant advantages in cancer management; accurate monitoring and localized intervention are essential aspects of this approach [23]. Nonetheless, it is worth mentioning that each nanoparticle system has inherent advantages for certain applications. Informed by the functionalized MNPs, this work highlights previously reported AuNPs, SeNPs, and GdNPs, describing their multifunctional properties and great potential for drug delivery applications [24,25,26,27,28]. We further summarized recent advancements on selenium nanoparticle-based drug delivery [29].

For the last 10 years, GdNPs have developed from simple MRI contrast agents to complex multifunctional theranostic platforms. Recent investigations have demonstrated their successful integration into diverse biomedical applications, including targeted drug delivery, multimodal imaging, and enhancement of radiation therapy through photodynamic therapy. Notwithstanding great progress, there are still several important bottlenecks in GdNPs design and clinical translation in drug delivery and theranostics. These gaps and challenges include (1) long-term toxicity and biodistribution issues, (2) clinical translation constraints, (3) limiting targeting efficiency, (4) stability and scalability issues, and (5) an urgent need for systematic comparative studies.

This review fills that gap by presenting a comprehensive and integrated analysis that exceeds prior reviews across multiple areas: synthesis-to-application pathways, critical functionalization strategies, mechanistic views of therapeutic actions, balancing challenges, and a perspective on clinical translation. We summarize the synthesis of GdNPs, biological targets to which GdNPs are targeted, explain their mechanisms of action, describe the major obstacles and limitations to clinical translation, and provide an opportunity to review existing articles to highlight technical, biological, and regulatory issues to overcome. Finally, we highlight emerging trends and future directions expected to inform the advancement of the GdNP nanomedicine.

### 1.3. Gadolinium (Gd)

Gd, a group IIIB lanthanide, is among the most abundant rare earth elements (REEs) found in the Earth’s crust [30]. The chemical behavior of most lanthanides is fairly similar, which explains why, in all, it took more than a century to split them into individual elements. The magnetic properties of Gd, in particular, are unique and explain many of its applications [31]. Element 64 is located halfway through the lanthanide 4f series, and the trivalent Gd^3+^ ion can organize its seven 4f electrons in a half-filled shell with all spins parallel, a magic member, which represents the maximum possible number within the lanthanide series. Although europium also possesses seven 4f electrons in its neutral state, Eu^3+^ lacks unpaired electrons and consequently does not exhibit comparable magnetic behavior. The unusually high number of unpaired electrons gives Gd (III) the largest possible total spin S = 7/2, and a correspondingly very large spin magnetic moment. This characteristic can be used to improve the performance of permanent magnets. The 4f shell of lanthanides has electron binding energies in the valence range, making it possible to chemically vary the 4f occupation, but the compact 4f radial size is typical of the outermost core electrons and prevents most 4f electrons from directly participating in bond formation [32].

However, because the orbital angular momentum of Gd^3+^ is effectively zero, its total magnetic moment arises almost entirely from electron spin. As a result, the experimentally observed and theoretically predicted magnetic moments of Gd^3+^ (approximately 7.90 and 7.94, respectively) place it below several other lanthanides in overall magnetic strength, despite its high spin contribution [33].

Unlike most other REEs, Gd exhibits exceptionally strong paramagnetic behavior [34], a property that underpins its widespread use as a “contrast agent” in MRI. When Gd accumulates in tissues, it significantly shortens *T*1 relaxation times, thereby enhancing signal intensity (brightness) on T1-weighted MRI scans [35]. The MRI signal comes from certain nuclear spins, such as the ubiquitous protons. That nuclear spin system, however, is heated by the radiofrequency field used for magnetic resonance. This heating typically weakens the MRI signal [21]. The large electronic magnetic moment of Gd (III) helps couple the nuclear spin systems to the “lattice” and keep it cool. This is called nuclear spin–lattice relaxation. To avoid health hazards, because Gd^3+^ is somewhat toxic, the Gd ion is surrounded by chelating ligands that prevent it from entering tissues [36]. New ligands are under development to improve safety [37].

Glycosaminoglycans (GAGs) are the primary contributors to the fixed charge density (FCD) of cartilage and are among the first components to be depleted during the early stages of arthritic disease. Beshir et al. hypothesized that, similar to sodium ions (Na^+^), the negatively charged contrast agent Gd-DTPA^2−^, and the resulting changes in proton T_1_ relaxation, could be used to quantify tissue-fixed charge density (FCD) and thereby estimate the GAG concentration. For example, when complexed with the chelating agent, diethylenetriamine penta-acetic acid (DTPA), Gd-DTPA^2^ affects the *T*1 MRI signal. Because of its charge, Gd-DTPA^2−^ has been used to measure the FCD for cartilage, which correlated with FCD measurements of explanted cartilage made using other techniques [38] as well as with the dynamic modulus and the sulfated glycosaminoglycan (GAG) content of bioreactor cultured cartilage [39]. Further, Gd-DTPA^2−^ has been used to measure the FCD of engineered cartilage constructs consisting of chondrocytes cultured in a poly(ethylene oxide) diacrylate hydrogel [40,41]. In another application, Gd-DTPA^2−^ has been used to label albumin to allow the quantification of protein concentration profiles using MRI [42]. In contrast to Gd-DTPA^2−^, iron oxide serves as a superparamagnetic MRI contrast agent, which shortens *T*2 relaxation times. Fe_3_O_4_ NPs have been used to label stem cells for in vivo MRI detection [43].

Beyond diagnostic imaging, the unique magnetic properties of Gd-based materials enable the generation of localized magnetic microenvironments. These microenvironments can be exploited to fabricate non-contact biological scaffolds, thereby supporting tissue engineering, 3D cell culture, and regenerative medicine applications [44,45,46].

Gd-containing compounds also contribute to bone-related processes by suppressing osteoclast activity, stimulating the differentiation of stem cells into osteoblasts, and promoting vascular development, all of which support osteogenesis [47,48]. Like other REEs, Gd can enhance the antimicrobial activity of carrier materials by modulating their surface charge and improving their photothermal properties [49].

In oncology, Gd-based platforms offer multiple therapeutic and diagnostic capabilities. They serve as effective contrast agents for tumor visualization and as nanocarriers for targeted drug delivery. Once localized at the tumor site, these materials can disrupt mitochondrial integrity, interfere with ATP production, and trigger oxidative stress, collectively driving tumor cell death and enabling multimodal anticancer action [50,51,52]. Gadolinium oxide (Gd_2_O_3_) in particular has demonstrated broad utility, including applications in drug delivery, cancer cell targeting and imaging, therapeutic intervention, cell labeling and tracking, and biosensing [53].

In addition to its biological applications, Gd plays a significant role in magnetic cooling technologies. In this process, known as the magnetocaloric effect, magnetic material increases in temperature when exposed to an external magnetic field due to the alignment of its magnetic dipoles. Conversely, when the magnetic field is removed under adiabatic (thermally isolated) conditions, the material undergoes cooling. By modulating the applied magnetic field and controlling thermal insulation, entropy is exchanged between the electronic spin system and the lattice degrees of freedom, enabling refrigeration [54].

### 1.4. Gadolinium Nanoparticles (GdNPs)

GdNPs have been shown to be a versatile platform for biomedical imaging and theranostic applications. GdNPs can be synthesized using a range of chemical, physical, and biological approaches, yielding diverse nanostructures such as gadolinium chloride [55,56,57,58,59], gadolinium oxide (Gd_2_O_3_) [60], gadolinium fluoride (GdF_3_) [61], and hybrid or core–shell nanoparticles [62]. Their physicochemical characteristics, particle size, morphology, crystallinity, and surface charge critically influence magnetic relaxivity, colloidal stability, biodistribution, and cellular uptake [53,63]. In many cases, nanoscale Gd formulations exhibit enhanced longitudinal (*T*1) relaxivity compared with molecular chelates, attributed to increased rotational correlation times and higher local Gd density [64].

Surface functionalization plays a pivotal role in improving the biocompatibility and multifunctionality of GdNPs. Coatings based on polymers, silica, peptides, lipids, or biomolecules are frequently employed to stabilize nanoparticles, reduce Gd ion leakage, and enable active targeting toward specific biological markers [65,66,67]. Through rational ligand design, GdNPs have been developed as multifunctional platforms capable of MRI contrast enhancement, targeted drug delivery, photothermal therapy, and radiosensitization, supporting their application in theranostic systems [68,69,70].

Although GdNP performance is promising, their safety and long-term fate are critical considerations for clinical translation. The release of Gd agents and bioaccumulation, as well as systemic toxicity, especially in patients with renal insufficiency, might be an issue [71,72]. Thus, current research is focusing on the development of new nanoparticle formulations, biodegradable coatings, and comprehensive in vivo assessments for better pharmacokinetics and clearance [53,73]. In summary, GdNPs constitute a strong and versatile nanotechnology platform for emerging diagnostic and therapeutic applications. Advancements in synthesis methods, surface engineering, and biological evaluation are anticipated to enhance their applications in precision medicine and targeted drug delivery paradigms [74].

## 2. Chemistry of GdNPs

This chemical versatility enables GdNPs to serve as safe, traceable, and highly adaptable nanocarriers, bridging diagnostic imaging with targeted and controlled therapeutic delivery. The drug-delivery chemistry of GdNPs relies on precise control of inorganic core composition, surface functionalization, and stimulus-responsive conjugation strategies.

From a drug delivery perspective, the chemistry of GdNPs is carefully designed to integrate high magnetic performance with efficient cargo loading, targeting, and controlled release. Gd is typically incorporated as Gd^3+^ within chemically stable inorganic matrices such as gadolinium oxide, fluoride, or hybrid organosilicate frameworks, which act as nanocarriers while minimizing the dissociation of free Gd^3+^ ions during systemic circulation [72,73,74]. These solid matrices provide structural robustness, protect encapsulated therapeutics, and enable simultaneous imaging and therapy, positioning GdNPs as ideal theranostic platforms.

Synthesis: GdNPs are not formed spontaneously from elemental Gd but are synthesized through controlled chemical processes that convert gadolinium ions (Gd^3+^) into nanoscale solid materials. Typically, a soluble gadolinium salt such as gadolinium chloride or nitrate is dissolved in aqueous or organic media, where it dissociates into Gd^3+^ ions. These ions are then reacted with precipitating agents (e.g., hydroxide, fluoride, or phosphate sources) under carefully controlled pH, temperature, and concentration. When supersaturation is reached, nucleation occurs, forming small crystalline clusters that grow into nanoparticles such as Gd_2_O_3_, GdF_3_, or GdPO_4_. Careful control of nucleation and growth is essential because particle size, crystallinity, and morphology strongly influence magnetic relaxivity and biomedical performance [75].

Stabilization: To maintain nanoscale size and prevent aggregation, stabilizing agents like polymers [76], surfactants [77], peptides [25], or silica coatings are added during or after synthesis. Common modes of fabrication include co-precipitation, hydrothermal/solvothermal synthesis, and thermal decomposition, which offer unique benefits regarding uniform size and structural integrity [78]. For numerous biomedical applications, Gd can be introduced or doped in stable nanomatrices (e.g., silica or iron oxide) to reduce the release of free Gd^3+^ ions, which can raise toxicity concerns. Subsequent surface functionalization enhances biocompatibility, targeting capability, and drug loading efficiency, thereby transforming Gd from a simple paramagnetic ion into a multifunctional theranostic nanoplatform [75].

**Figure 1:**
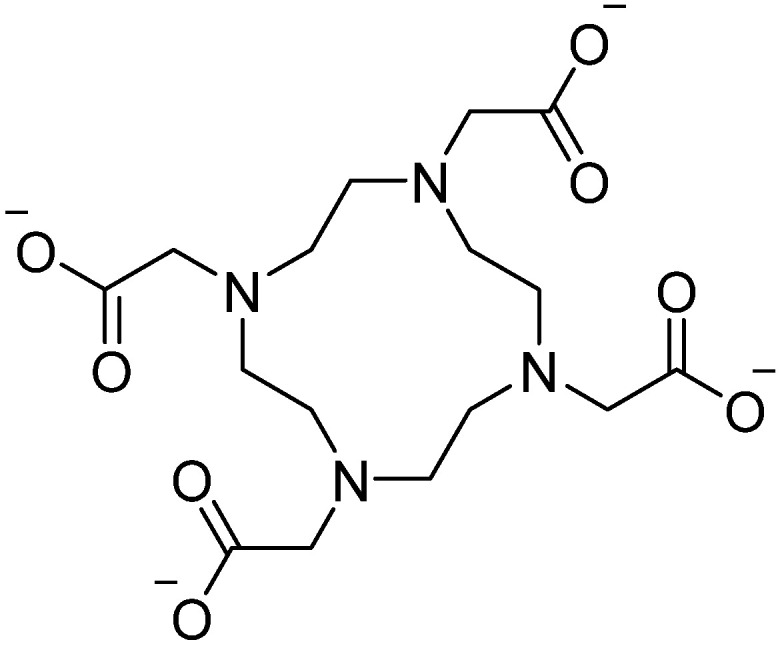
Chemical structure of octadentate polyaminocarboxylate.

Coordination: GdNPs, like other members of the lanthanide family, function as a hard Lewis acid and therefore favor predominantly ionic interactions with coordinating species [79]. All GdNP chelates currently used in clinical and therapeutic applications are derived from octadentate polyaminocarboxylate ligand frameworks (Figure 1).

These ligands coordinate strongly to the Gd^3+^ ion, occupying eight of its nine preferred coordination sites. The remaining coordination position is typically filled by a water molecule, which plays a key role in modulating proton relaxation in magnetic resonance imaging. Chelation involves the formation of a stable five-membered rings by the coordination of nitrogen and carboxylate donor atoms to the metal center. These chelate rings are generated through metal–ligand ionic interactions and differ fundamentally from classical five-membered organic heterocycles, such as furan, which are stabilized by covalent bonding rather than metal coordination [79].

The coordination chemistry of GdNPs and size regulation will also affect intracellular trafficking, drug release, and clearance. Ultrasmall and biodegradable GdNP formulations have been shown to facilitate renal clearance following drug delivery, reducing long-term tissue retention and toxicity risks [72,80]. Critically, the potent *T*1 relaxivity of Gd enables real-time MRI tracking of nanoparticle biodistribution and drug-delivery efficiency, providing direct feedback on therapeutic localization and treatment response [64,72,73,74].

Surface chemistry: The surface chemistry of GdNPs is central to drug loading and delivery efficiency. Therapeutic agents, including chemotherapeutic drugs, radiosensitizers, nucleic acids, and photosensitizers, can be physically encapsulated within porous structures or chemically conjugated to surface functional groups via covalent or electrostatic interactions [72,81,82]. Carboxylate-, phosphonate-, and silane-based chemistries are commonly employed to anchor drugs either directly or through stimulus-responsive linkers. These linkers can be engineered to respond to acidic pH, redox gradients, or enzymatic activity, enabling site-specific drug release in tumor microenvironments or intracellular compartments [73,83].

Chemical functionalization further enables multimodal and targeted drug delivery. Polymeric coatings, particularly polyethylene glycol (PEG), are frequently grafted onto GdNP surfaces to enhance colloidal stability, prolong circulation time, and reduce nonspecific protein adsorption [71,72,74]. Targeting ligands, such as peptides, antibodies, or small molecules, can be conjugated via orthogonal surface reactions, promoting receptor-mediated uptake and increasing drug accumulation at diseased sites [74,84,85,86]. This modular surface chemistry allows independent optimization of pharmacokinetics, targeting specificity, and therapeutic payload.

## 3. Absorption, Distribution, and Elimination of GdNPs

GdNPs have recently gained importance as an alternative to low-molecular-weight Gd chelates by means of increased relaxivity, increased imaging sensitivity, and potential for multifunctional theranostic use [75,87,88]. In contrast to molecular Gd complexes, GdNPs display unique pharmacokinetic profiles determined by their nanoscale dimensions, surface chemistry, and structural organization. Given this, knowledge of these pharmacokinetic profiles should be complemented to optimize imaging performance whilst minimizing toxicity and long-term tissue retention.

The selected studies summarized in this section demonstrate that the pharmacokinetics of Gd-based agents, whether formulated as conventional chelates or nanoscale constructs, are governed by a complex interplay of molecular stability, physicochemical properties, and biological handling.

The pharmacokinetic behavior of Gd is defined by its absorption, distribution, metabolism, and excretion (ADME), which differ markedly from those of small-molecule Gd agents [89,90]. Following intravenous administration, GdNPs rapidly interact with plasma proteins and elements of the mononuclear phagocyte system, influencing circulation half-life and biodistribution [91].

Physicochemical parameters such as particle size, shape, surface charge, and surface functionalization, including polymeric, peptide-based, or ligand-directed coatings, play critical roles in determining organ accumulation, particularly in the liver, spleen, kidneys, and tumor tissues [92,93,94]. Strategic surface engineering has been shown to modulate pharmacokinetics by prolonging systemic circulation, enhancing renal clearance, or promoting active targeting of diseased tissues [95,96].

Excretion pathways are a central consideration in the pharmacokinetics of GdNPs, especially given increasing concerns regarding Gd retention and associated toxicities [57,97,98]. Ultrasmall GdNPs with hydrodynamic diameters below the renal filtration threshold may undergo efficient renal clearance, whereas larger or aggregated systems are more likely to be eliminated through hepatobiliary routes, increasing the risk of long-term tissue accumulation [84,99].

Furthermore, the thermodynamic and kinetic stability of Gd within the nanoparticle matrix is a key determinant of biosafety, as the dissociation and release of free Gd^3+^ ions have been linked to adverse outcomes such as nephrogenic systemic fibrosis and persistent tissue deposition [100,101,102]. Consequently, rigorous pharmacokinetic evaluation remains essential for the safe and effective translation of GdNPs into clinical imaging and theranostic applications.

Neburkova et al. [103] reported that gadolinium can accumulate in the brain after GBCA administration. They demonstrated that trace amounts of Gd^3+^ released from GBCAs form Gd^3+^ ferritin nanoparticles at nanomolar concentrations under physiological conditions. Structural analysis showed that Gd^3+^ binds to the surface of the ferritin oxyhydroxide core, and this process is driven by the kinetic instability of the GBCA rather than its thermodynamic stability. The formation of these Gd^3+^–ferritin complexes in serum may partly explain the increased *T*1 signal intensity observed in ferritin-rich brain regions after repeated GBCA exposure.

Gd deposition in patients with normal renal function was first described by Kanda et al. [58], who observed T1-weighted hyperintensity in the globus pallidus and dentate nucleus following multiple doses of linear GBCAs. Later studies confirmed that these signal changes are associated with Gd retention and raised concerns about potential long-term neurotoxicity [98,104,105]. Although macrocyclic GBCAs appear to result in lower brain accumulation than linear agents, they are not entirely free from deposition [106,107,108,109,110,111].

The kidney is also recognized as a primary site of Gd retention in both humans and animal models. Differences in renal persistence among macrocyclic agents have been reported, but it remains uncertain whether MRI can accurately detect residual renal Gd or determine whether it is retained as intact GBCA or transformed chemical species.

**Figure 2:**
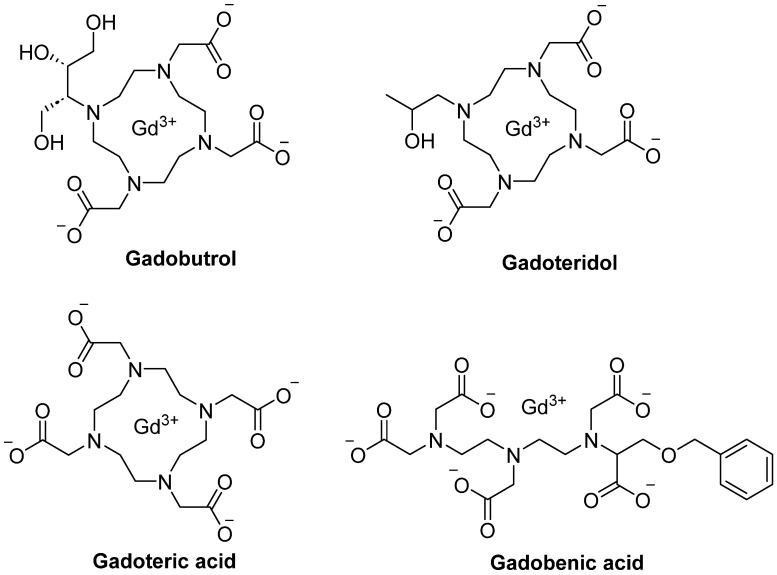
The chemical structures of gadoteric acid, gadoteridol, gadobutrol, and gadobenic acid.

Mounting evidence indicates that repeated exposure to Gd-based contrast agents (GBCAs) leads to Gd deposition in the brain and peripheral organs long after administration, yet the biodistribution and chemical form of retained Gd remain poorly understood. Le Fur et al. (2023) compared the pharmacokinetics, distribution, and speciation of four GBCAs, gadoterate, gadoteridol, gadobutrol, and gadobenate, in healthy rats using MRI, mass spectrometry, elemental imaging, and electron paramagnetic resonance spectroscopy, alongside analysis of human kidney specimens [112]. They performed their in vivo investigation using 32 rats that received a dose of gadoteric acid, gadoteridol, gadobutrol, or gadobenic acid (2.0 mmol/kg) for 10 consecutive days. The structures of these compounds are illustrated in Figure 2.

**Figure 3:**
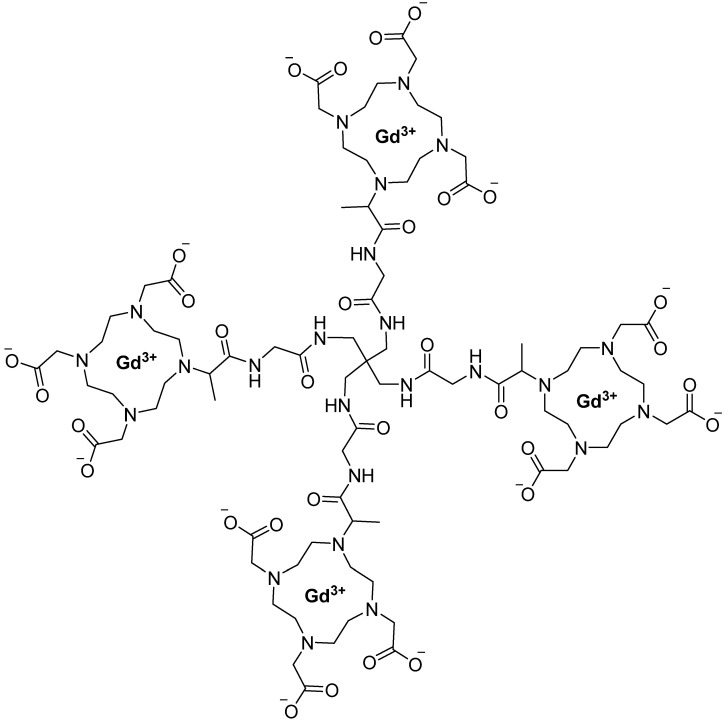
The chemical structures of tetrameric gadoquatrane.

GBCA-naive rats served as controls in a longitudinal study in which three-dimensional T1-weighted UTE MRI and R2* maps of the kidneys were acquired at 3, 17, 34, and 52 days post-injection, and gadolinium levels in 23 organs, tissues, and fluids were quantified by mass spectrometry at 17 and 52 days, with renal distribution and cortical speciation further characterized by elemental imaging and EPR spectroscopy; statistical analyses included ANOVA, Kruskal–Wallis, response profile analysis, and Pearson correlation, and Gd concentrations in multiple organs, including the kidney cortex and medulla, were reported in nmol/g wet tissue [112]. Analysis of human kidney samples revealed detectable Gd weeks after GBCA exposure, with higher levels in patients receiving multiple doses, supporting prolonged renal retention; among the agents evaluated, gadoteridol showed the most efficient elimination, followed by gadoterate, gadobutrol, and gadobenate, and although the kidneys exhibited the highest Gd concentrations overall, significantly lower renal cortex retention was observed for gadoteridol compared with gadobutrol and gadobenate at 52 days post-injection, with no evidence of renal injury. R2* mapping proved more sensitive than T1-weighted MRI for detecting renal Gd and correlated strongly with ex vivo Gd concentrations, whereas T1-weighted imaging failed to detect high retained levels, and EPR spectroscopy confirmed that Gd was primarily retained as intact chelates [112]. In a separate investigation, Guenther et al. (2025) evaluated the pharmacokinetics of gadoquatrane (Figure 3), a tetrameric, macrocyclic, extracellular Gd-based MRI contrast agent with high relaxivity and kinetic stability currently in Phase 3 clinical development, in female cynomolgus monkeys and compared it with established macrocyclic agents using analytically distinguishable lanthanide analogs; following intravenous administration, gadoquatrane demonstrated a rapid, multiphasic plasma decline characterized by fast distribution, efficient systemic elimination, and a slow terminal phase, yielding an effective half-life of approximately 1 h, along with a low volume of distribution and clearance values consistent with extracellular, renally eliminated contrast agents [113].

Gadoquatrane was rapidly and almost exclusively excreted via the kidneys, with approximately 97% of the administered dose recovered in urine within 24 h. Residual urinary excretion persisted at very low levels over extended periods, while tissue retention was minimal and decreased substantially over time, with the highest concentrations observed in the kidney cortex and markedly lower levels in the skin and brain. Tissue concentrations declined between early and late sampling time points, and no metabolic degradation products were detected, confirming the compound’s metabolic stability. The pharmacokinetic behavior and tissue distribution of gadoquatrane in nonhuman primates were comparable to those of established macrocyclic contrast agents, with no evidence of increased long-term Gd retention [113].

**Figure 4:**
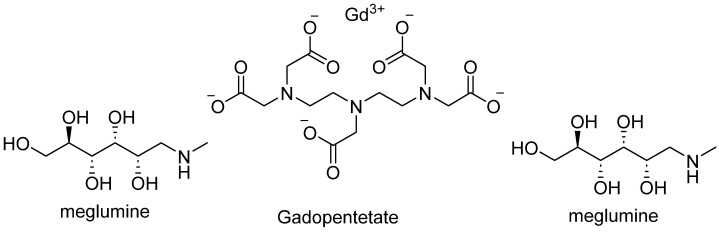
The chemical structures of gadopentetate dimeglumine.

Moreover, the excretion of gadopentetate dimeglumine (Figure 4) into human breast milk was evaluated in 20 lactating women following intravenous administration of a standard clinical dose [114]. Serial milk samples collected over 24 h demonstrated that Gd transfer into breast milk was minimal, with a cumulative excretion of less than 0.04% of the administered dose in all subjects. The mean excreted fraction was approximately 0.009%, corresponding to absolute amounts in the micromolar range. Based on these findings, the estimated oral exposure of a nursing infant would be more than two orders of magnitude lower than the approved intravenous dose for neonates, indicating negligible clinical risk. These results suggest that routine interruption of breastfeeding for 24 h after gadopentetate dimeglumine administration may not be necessary.

Furthermore, Hahn et al. (2009) reported a clinical study with a focus on the pharmacokinetics and safety of gadobutrol (Figure 2), an extracellular Gd-based MR contrast agent in pediatric patients aged 2–17 years undergoing routine contrast-enhanced MRI or MR angiography [115]. Across age groups, gadobutrol pharmacokinetics were well described by a two-compartment model with renal elimination from the central compartment. Body weight-normalized clearance and volume of distribution values were consistent with those observed in adults and showed predictable variation with body weight, while age itself did not independently influence pharmacokinetic parameters. Simulated plasma gadolinium (Gd) concentrations after a weight-based (dose/kg) administration were within expected ranges across pediatric weights, indicating similar systemic exposure in children and adults.

Most doses of gadobutrol were eliminated through the kidneys, with approximately 77% of the dose being excreted in the urine within 6 h, confirming rapid renal clearance in pediatric patients. The agent was tolerated well and only a small percentage of patients were affected by mild drug-related adverse reactions. Disparities in gadobutrol pharmacokinetics in children are largely associated with body weight rather than age and support the use of the standard weight-based adult dose (0.1 mmol/kg) in pediatric patients of 2–17 years old without titrating the dose [115].

In general, macrocyclic GBCAs exhibit favorable kinetic stability and rapid renal elimination, but mounting evidence indicates that measurable amounts of Gd can persist in the brain, kidneys, and other tissues for extended periods, even in individuals with normal renal function. The kidney emerges as a key reservoir for residual Gd, with agent-dependent differences in retention and clearance that are not reliably captured by conventional *T*1-weighted MRI. Additionally, results in the generation of Gd^3+^–ferritin nanoparticles reveal that in vivo the possibility of secondary nanoparticle-like species can arise, which challenges the assumptions about Gd speciation and toxicity. Therefore, rigorous pharmacokinetic and speciation investigations, advanced imaging and analytical methods, and rational material design should be the prerequisites for the safe development and clinical translation of the next-generation GdNPs and contrast agents [74,116,117,118].

## 4. GdNPs in Drug Delivery

### 4.1. Gadolinium (Gd)

GdNPs have emerged as multifunctional platforms in drug delivery due to their unique electronic structure and high atomic number. GdNPs serve as valuable nanocarrier design tools, as the incorporation of Gd allows precise control over nanoparticle size, morphology, and surface charge. Particle size critically influences pharmacokinetics, tumor accumulation via the enhanced permeability and retention effect, and intracellular trafficking, while surface charges modulate cellular uptake, endosomal escape, and interactions with serum proteins. Positively charged GdNPs often exhibit enhanced cellular internalization, whereas negatively charged or neutral surfaces can improve colloidal stability and circulation time [74,92,93]. Herein, we summarize the different roles of GdNPs in drug delivery systems.

### 4.2. Used as Photo-Thermal Nanoparticles and Work Through Increasing Energy

On one hand, GdNPs can be used in photothermal or energy-amplifying agents where they facilitate local energy generation under external stimuli like near-infrared (NIR) light, X-ray irradiation, or radiofrequency fields. Excited GdNPs can convert the absorbed energy to heat. The localized hyperthermia caused then promotes tumor cell death and the improvement of therapeutic efficacy by increasing membrane permeability and facilitating drug release. GdNPs can also be used as radiosensitizers in radiation-induced energy deposition and ROS generation in tumor tissues.

Another great example is AGuIX^®^ nanoparticles [119]. AGuIX^®^ nanoparticles (Activation and Guiding of Irradiation by X-ray) are ultrasmall (~3–5 nm) polysiloxane-based GdNPs designed as theranostic agents for MRI-guided radiotherapy. Structurally, they consist of a rigid polysiloxane matrix covalently grafted with gadolinium chelates (typically DOTA-type ligands), enabling high Gd payload per particle while maintaining strong kinetic stability. Their small hydrodynamic diameter permits renal clearance, reducing long-term tissue retention. Functionally, AGuIX^®^ nanoparticles act as *T*1 MRI contrast agents due to the paramagnetic properties of Gd^3+^ and simultaneously serve as radiosensitizers: the high atomic number of Gd enhances local dose deposition during irradiation, increasing ROS generation and amplifying tumor cell damage. These particles preferentially accumulate in tumors via the enhanced permeability and retention (EPR) effect and have progressed into clinical evaluation for image-guided radiotherapy in brain metastases and other solid tumors.

When these compounds are engineered to absorb NIR or radiofrequency energy, Gd-containing nanostructures, including gadolinium oxide nanoparticles, Gd-doped hybrid nanoparticles, and Gd-integrated nanocomposites, can generate heat through non-radiative relaxation processes [75,84]. This photothermal effect permits spatially controlled hyperthermia that can directly induce cancer cell death but can also facilitate drug release through the increased permeability of the membrane, endosomal escape, and increased drug release from thermoresponsive carriers [120,121]. The high atomic number and paramagnetic properties of Gd also facilitate the coupling of photothermal therapy with MRI-guided treatment, making it to be monitored for nanoparticle accumulation and therapeutic response in real time [49].

In drug delivery applications, GdNPs can generate photothermal energy when exposed to light. This reaction can initiate controlled drug release and improve therapeutic outcomes. Local heating also disrupts tumor vasculature, increases intratumoral delivery of drugs, and sensitizes the cancer cell to chemotherapy or radiotherapy by heightening the intracellular stress and impairing DNA repair pathways [51,52,122,123,124,125]. Furthermore, Gd-based nanostructures have shown attractive performance also in photothermal therapy (PTT) and multifunctional therapy systems. Gd_2_O_3_ nanoparticles and Gd-doped hybrid nanostructures, to provide another example, can absorb external energy sources; these may be radiofrequency (RF) fields, near-infrared (NIR) light, etc., allowing for efficient non-radiative relaxation and localized generation of heat. Photothermal conversion facilitates triggering hyperthermia, which is capable of injuring the tumor cells for improved therapeutic action [126]. As a further analysis, a GdPO_4_/CS/Fe_3_O_4_ composite scaffold that was activated by 808 nm NIR laser achieved remarkable photothermal performance with a 10 min increased temperature from 20 °C to ~47.7 °C stimulating tumor cell apoptosis. Apart from the ablation of the tumor, the scaffold showed regenerative potential, with BV/TV increasing to approximately 61% pointing to improved bone regeneration. Their findings point to gadolinium-based nanomaterials as potential dual-functional platforms for multi-instrument, multimodal strategies including, but not limited to, photothermal tumor therapy and drug delivery with the aim of facilitating tissue regeneration in breast cancer bone metastases [127,128].

Zhao et al. (2020) [128] developed multifunctional GdPO_4_/CS/Fe_3_O_4_ composite scaffolds for simultaneous photothermal tumor ablation and bone regeneration in breast cancer bone metastasis by hydrothermally synthesizing hydrated GdPO_4_·H_2_O nanorods (≈80 nm width, 3 µm length) at 180 °C for 24 h and integrating them with Fe_3_O_4_ nanoparticles (~20 nm) into a chitosan matrix via freeze-drying, yielding highly porous scaffolds with interconnected macropores (~100 µm). Under 808 nm near-infrared (NIR) irradiation (4.6 W/cm^2^), the GdPO_4_/CS/Fe_3_O_4_ scaffolds rapidly increased in temperature from 20 °C to 47.7 °C within 10 min, whereas control scaffolds lacking Fe_3_O_4_ remained below 29 °C; in vivo, local temperatures reached ~45.4 °C within 20 s, inducing significant tumor apoptosis and marked tumor diameter reduction after 14 days of treatment. Beyond photothermal efficacy, the scaffolds demonstrated strong osteogenic capacity, with minimal ion release after 120 h of degradation (1.58 µM Gd^3+^ and 1.37 µM Fe, within safe biological limits). In rat calvarial defect models (5 mm defects), new bone volume/tissue volume (BV/TV) reached 58.15 ± 3.45% (GdPO_4_/CS) and 61.23 ± 2.12% (GdPO_4_/CS/Fe_3_O_4_), significantly higher than the blank control (10.01 ± 1.86%) and CS alone (19.16 ± 2.87%), with mineralization rates of 5.62 ± 0.38 and 6.19 ± 0.31 µm/day, respectively, more than double that of CS (2.57 ± 0.41 µm/day). Mechanistically, controlled Gd^3+^ release promoted M2 macrophage polarization and activated the BMP-2/Smad/RUNX2 pathway, enhancing VEGF-mediated angiogenesis and osteogenic differentiation, thereby integrating localized photothermal therapy with accelerated vascularized bone regeneration in a single dual-functional platform for treating breast cancer bone metastases [128].

Cui et al. (2024) [129] integrated MRI contrast capability, microwave-induced immunogenic cell death, and checkpoint blockade to achieve synergistic thermotherapy and immunotherapy with strong tumor inhibition and favorable biosafety. They reported the design of a microwave-responsive Gd metal–organic framework (Gd-MOF) nanosystem (Gd/MPC) for MRI-guided thermotherapy combined with PD-1 checkpoint blockade immunotherapy. The nanosystem was fabricated by first synthesizing Gd-MOF (Gd/M), followed by loading with the anti-PD-1 antibody (aPD-1) with a quantified loading proportion of 28.23%. A phase change material (1-tetradecanol, melting point 38–40 °C) was incorporated at a mass ratio of approximately 28%, enabling temperature-triggered drug release. Finally, SCC7 cancer cell membranes were coated onto the surface, increasing the hydrodynamic diameter from approximately 163 nm (Gd/MP) to 182 nm (Gd/MPC), consistent with a 5–10 nm membrane layer. Under microwave irradiation (0.6 W/cm^2^), a 1 mg/mL Gd/MPC solution exceeded 45 °C within 5 min, whereas the medium alone only reached ~40 °C. At higher microwave power (up to 1.0 W/cm^2^) or concentration (up to 4 mg/mL), heating showed clear power and concentration dependence. Importantly, even with 15 mm pork tissue coverage, the temperature still increased efficiently, demonstrating strong tissue penetration. In vitro, microwave-treated Gd/MPC raised local temperatures to near 50 °C, completely suppressing SCC7 proliferation over 48 h, while Gd/MPC alone (100–200 µg/mL) showed minimal cytotoxicity, confirming thermal-specific tumor killing [129].

Under the in vivo experiment, C3H mice bearing SCC7 tumors (~50 mm^3^ at treatment initiation) were divided into seven groups (*n* = 5 per group). After intravenous injection, microwave irradiation (0.6 W/cm^2^, 10 min) was applied 4 h post-injection, coinciding with peak tumor accumulation detected by fluorescence and MRI. Tumor temperatures in the Gd/MPC + MW group approached 55 °C, compared to ~50 °C with microwave alone. By day 12, the Gd/MPC + MW group showed the strongest tumor suppression, significantly outperforming PBS, Gd/M, and aPD-1 alone groups. Quantitatively, the MOF delivery system improved antitumor efficacy by ~26% compared to free aPD-1; microwave hyperthermia contributed ~15% additional inhibition; incorporation of a PD-1 increased efficacy by 59% compared to Gd/M alone; and homologous membrane coating added a further 15% improvement [129].

Jiang et al. (2021) [130] demonstrated a highly effective, low-toxicity MRI-guided photothermal chemotherapy system with strong tumor imaging, controlled drug release, and synergistic antitumor efficacy for TNBC. They developed a multifunctional theranostic nanoplatform based on Gd-encapsulated carbon dots (Gd@CDs) for magnetic resonance imaging (MRI)-guided photothermal chemotherapy of triple-negative breast cancer (TNBC). The Gd@CDs were synthesized via a hydrothermal method using 3,4-dihydroxyhydrocinnamic acid, ethylenediamine, and GdCl_3_ at 200 °C for 5 h. HRTEM analysis showed spherical nanoparticles with an average core diameter of 2.58 nm and a lattice spacing of 0.16 nm, while dynamic light scattering indicated a hydrodynamic size of 308.8 nm due to aggregation. ICP-MS confirmed a Gd content of 0.459%. The carbon dots exhibited excitation-dependent fluorescence with a maximum emission at 437 nm (excitation 360 nm), a fluorescence lifetime of 3.58 ns, and a quantum yield of 26.84%. Importantly, Gd@CDs demonstrated a 6.53-fold higher longitudinal relaxation rate (R_1_) compared to clinical Gd-DTPA at the same Gd concentration, indicating superior *T*1 MRI contrast capability. In vitro cytotoxicity tests showed >90% viability of 293T cells even at 1 mg/mL after 24 h, and in vivo studies in mice (0.3 and 3 mg/kg injections for 16 days) showed no significant changes in liver or kidney biochemical markers, confirming good biocompatibility [130].

The therapeutic platform was constructed by loading doxorubicin (Dox) and the NIR photothermal agent IR825 onto Gd@CDs, forming Dox@IR825@Gd@CDs. Drug loading efficiencies were 16.4% for Dox and 8.9% for IR825, with encapsulation efficiencies of 68.8% and 83.3%, respectively. Under acidic conditions (pH 4.92), 76.4% of Dox was released within 120 h, compared to 55.6% at pH 7.38, indicating tumor microenvironment-responsive release. Upon 808 nm laser irradiation (3 W, 5 min), a 1 mg/mL solution showed a temperature increase of 25.4 °C to approximately 57 °C, and maintained photothermal stability over five irradiation cycles. In vitro, combined photothermal chemotherapy reduced 4T1 cell viability to about 26% at 0.2 mg/mL under irradiation, and at 0.5 mg/mL more than 97% of cells were killed. In vivo, tumor-bearing mice treated with Dox@IR825@Gd@CDs plus NIR irradiation exhibited the strongest tumor suppression over 14 days, with tumor accumulation reaching 5.4% injected dose per gram (ID/g). No significant body weight loss or motor coordination impairment (rotarod test) was observed [130].

Table 1 elaborates on gadolinium-based nanoparticles that function with their photothermal properties and their energy modalities.

### 4.3. Surface, Size, and Charge Modification of GdNPs for Drug Delivery

GdNPs enhance drug delivery efficiency by enabling precise control of nanoparticle size and surface charge, two fundamental parameters that govern circulation behavior, tumor accumulation, cellular uptake, and intracellular trafficking [131]. The unique coordination chemistry of gadolinium ions (Gd^3+^) enables strong, stable interactions with chelating ligands, peptides, polymers, and inorganic matrices, facilitating the rational design of nanostructures with controlled size distributions and high structural stability [132,133]. By acting as a coordination center or crosslinking element, Gd contributes to the formation of compact, uniform nanoparticles, minimizing aggregation and ensuring reproducible drug loading and release characteristics.

The ability of GdNPs to modulate size and surface charge simultaneously may allow fine-tuning for the production of tailor-made drug delivery platforms with optimal pharmacokinetics, tumor selectivity, and enhanced intracellular drug delivery. Such a physicochemical flexibility, along with Gd’s intrinsic imaging and radiosensitizing properties, makes GdNPs promising multifunctional nanocarriers for advanced cancer drug delivery and theranostic purposes.

Control of nanoparticle size is particularly critical for optimizing in vivo drug delivery. GdNP-based systems can be engineered within the optimal size window of approximately 10–100 nm, which favors passive tumor targeting via the enhanced permeability and retention (EPR) effect while limiting premature renal clearance and excessive uptake by the mononuclear phagocyte system [131]. Smaller GdNPs exhibit improved tumor penetration and diffusion through dense tumor matrices, whereas moderately larger constructs benefit from prolonged blood circulation and higher drug payload capacity [74,132]. This size tunability allows Gd-based nanocarriers to be tailored for specific therapeutic objectives, including deep tumor penetration, sustained drug release, or combined diagnostic–therapeutic (theranostic) applications.

For example, Siribbal et al. (2024) [134] reported the hydrothermal synthesis of hollow Gd_2_O_3_ nanocarriers (~100–120 nm in diameter) using carbon nanospheres (~200 nm) as sacrificial templates, followed by calcination at 800 °C to generate porous hollow structures. The nanocarriers exhibited longitudinal and transverse relaxivities of r_1_ = 1.8 s^−1^·mM^−1^ and r_2_ = 5.3 s^−1^·mM^−1^ (1.41 T, 40 °C), confirming their potential as positive MRI contrast agents. To improve biocompatibility and reduce Gd^3+^ ion leaching, the particles were coated with citric acid (CA), which significantly enhanced cell viability. While bare Gd_2_O_3_ showed toxicity above 5 μg/mL, CA-capped nanocarriers maintained ~100–120% cell viability after 48 h, even at concentrations up to 100 μg/mL [134].

Drug loading and release studies were conducted using Congo red (CR) and sparfloxacin (SP). CA functionalization markedly improved loading capacity, increasing CR loading from 6.75% (bare) to 20% (CA-capped). Under physiological conditions (pH 7.4, 37 °C), bare particles released ~80% of CR within 8 h and nearly all within 24 h, whereas CA-coated carriers provided sustained release up to 96 h. For SP, CA-capped nanocarriers achieved 4% loading (vs. 1.78% for bare nanocarriers) and demonstrated prolonged antibiotic release for up to 168 h, maintaining effective antibacterial activity at concentrations as low as 0.03 mg/mL against both *E. coli* and *S. aureus*. Additionally, confocal microscopy confirmed time-dependent cellular internalization within 10 min to 24 h, highlighting the dual imaging and drug-delivery capability of the hollow Gd_2_O_3_ platform [134].

Surface charge modulation represents another key mechanism through which GdNPs improve drug delivery performance. GdNPs can readily be functionalized with positively or negatively charged ligands, such as amine-rich polymers, carboxylated coatings, or zwitterionic molecules, granting fine control over electrostatic interactions with biological environments [135]. The enhanced cellular internalization associated with positively charged GdNPs and their electrostatic attraction to negatively charged cell membranes and nucleic acids enable them to be particularly effective in delivering DNA, siRNA, mRNA, and nucleus-targeting chemo-therapeutic agents [63,136]. Cationic surface charge also destabilizes the membrane, promoting endosomal escape and increasing intracellular drug bioavailability.

Conversely, negatively charged or near-neutral GdNPs reduce nonspecific protein adsorption and opsonization in the bloodstream, thereby prolonging systemic circulation and improving tumor accumulation [131]. Charge-shielding strategies, such as PEGylation or charge-reversible surface coatings, can be combined with Gd-based cores to achieve stimulus-responsive charge switching neutral during circulation and positively charged within the acidic tumor microenvironment, thereby maximizing both safety and therapeutic efficacy [137]. Importantly, the ability to finely balance surface charge could also reduce off-target toxicity and minimize immune recognition.

In addition to engineering surface chemistry, particle size, and charge to optimize circulation time and cellular uptake, gadolinium-based nanoparticles (GdNPs) have been extensively functionalized with polymers, inorganic nanostructures, peptides, and therapeutic payloads to enhance drug loading efficiency, targeting, and controlled release. For example, polymer-coated composites, hollow and mesoporous Gd_2_O_3_ structures, cyclic peptide–Gd assemblies, and albumin-coated nanocarriers have demonstrated improved drug encapsulation, pH-responsive or sustained release, enhanced intracellular transport, or synergistic anticancer activity. Table 2 shows selected functionalized GdNP drug delivery platforms highlighting how polymer, peptide, inorganic, and albumin coatings enhance loading efficiency, targeting, and therapeutic outcomes.

Ganesh et al. (2025) [138] developed a rapamycin-loaded, chitosan-coated ZnO/TiO_2_/Gd_2_O_3_ nanocomposite (RNC) to enhance drug bioavailability and overcome multidrug resistance in non-small cell lung cancer (NSCLC) using A549 cells, with structural characterization confirming crystalline ZnO (wurtzite), Gd_2_O_3_ (cubic), and TiO_2_ (anatase) phases, and FTIR and EDX analyses verifying successful chitosan coating and rapamycin incorporation. Drug loading studies showed that 150 mg of the composite achieved 100% loading efficiency for 400 μg rapamycin, corresponding to 3.75 μg composite per 1 μg drug, substantially exceeding previously reported lipid–polymer systems (~67.8%); in vitro release experiments demonstrated complete diffusion of free rapamycin within 3 h, whereas the RNC enabled sustained and controlled release with 100% drug release over 6 h, indicating improved delivery and potential bioavailability [138]. Biologically, the RNC exhibited enhanced anticancer efficacy, with an LC_50_ of 31 μg/mL against A549 cells compared to 32 μg/mL for free rapamycin, while maintaining lower toxicity toward normal L929 cells (103 μg/mL for RNC vs. 100 μg/mL for rapamycin), demonstrating selective cytotoxicity; reactive oxygen species (ROS) levels increased markedly from 1221 AU (control) to 4592 AU (rapamycin) and further to 10,281 AU with RNC treatment, nearly threefold higher than the free drug. Flow cytometry revealed a reduction in viable cells from 95% (control) to 46.9% following RNC treatment, accompanied by increased early apoptosis (10.6%) and total cell death (19.4%), as well as pronounced G0/G1 cell cycle arrest (80.5%) compared with 65.9% for rapamycin alone, alongside elevated caspase-3 and p62 expression, confirming enhanced apoptosis and autophagy; collectively, the composite demonstrated synergistic anticancer activity mediated by controlled release, ROS amplification, apoptosis induction, and cell cycle arrest [138].

In another study, Babayevska et al. (2025) [139] synthesized crystalline Gd_2_O_3_ solid spheres (Gd_2_O_3__S) and hollow spheres (Gd_2_O_3__HS) via soft-chemistry methods, followed by ZnO coating and doxorubicin (Dox) loading, producing annealed solid spheres of ~120 nm (from ~170 nm precursors) and hollow spheres of ~150 nm with tunable shell thicknesses (20–50 nm); BET analysis revealed a higher surface area for Gd_2_O_3__HS (32.5 m^2^/g) than Gd_2_O_3__S (15.6 m^2^/g), directly enhancing drug loading capacity, and upon Dox adsorption, surface areas decreased to 1.9 m^2^/g (Gd_2_O_3__S@Dox) and 12.5 m^2^/g (Gd_2_O_3__HS@Dox), confirming successful loading. Drug adsorption was strongly pH- and temperature-dependent, reaching ~15% (Gd_2_O_3__S) and ~52% (Gd_2_O_3__HS) at pH 7.4 and 37 °C after 24 h, but increasing dramatically under mildly acidic conditions (pH 5.5, 37 °C) to 82.31% and 95.41%, respectively, highlighting the superior performance of hollow structures due to greater internal surface area; controlled release studies over 48 h showed pH-responsive behavior, with cumulative release at pH 7.4 plateauing at ~85% (Gd_2_O_3__S) and ~76% (Gd_2_O_3__HS) after 24 h, whereas acidic conditions (pH 5.5) resulted in lower release (~50% and ~27% after 48 h), indicating stronger drug retention in hollow spheres. Cytotoxicity assays (1–150 µg/mL, 24 h) demonstrated good biocompatibility of pristine Gd_2_O_3_ nanospheres toward MSU1.1 fibroblasts and HeLa cells (≈70–72% viability only at 150 µg/mL), while ZnO-containing systems exhibited increased toxicity at ≥100 µg/mL due to Zn^2+^ release and ROS generation; Dox-loaded hollow spheres (7 mg/mL loading solution) produced enhanced cytotoxicity at 100 µg/mL nanoparticle concentration. Photoluminescence analysis revealed strong visible emissions at 460, 484, 523, and 570 nm (maximum at ~460 nm under 273 nm excitation), supporting bioimaging capability, and confocal microscopy confirmed efficient cellular uptake and intracellular Dox release after 6 h at 50 µg/mL, demonstrating the theranostic potential of ZnO- and Dox-functionalized Gd_2_O_3_ hollow spheres for combined imaging and targeted cancer therapy [139].

**Figure 5:**
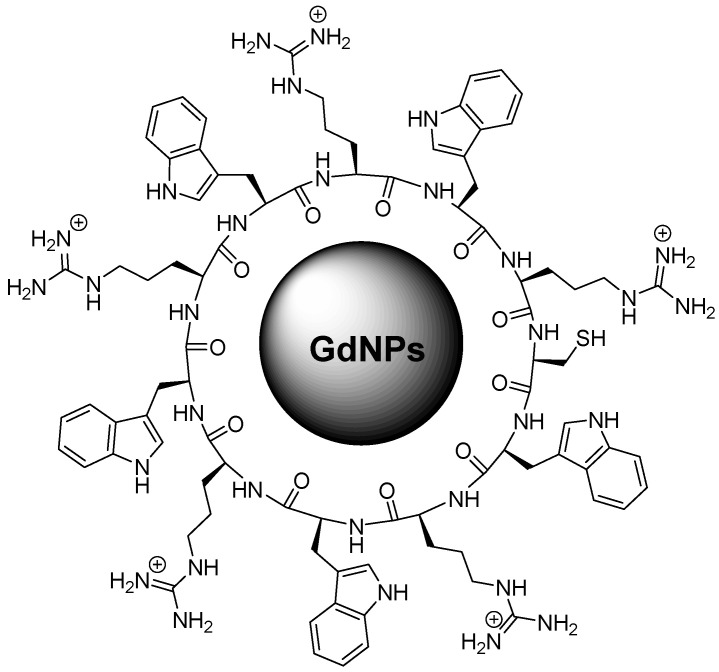
Structure of [(WR)_5_C]-GdNPs [25].

We reported that the 11-amino-acids cyclic peptide, [(WR)_5_C], containing five arginine, five tryptophan, and one cysteine residue, was used to generate GdNPs for enhanced intracellular delivery of small molecules and drugs (Figure 5). The peptide was synthesized via Fmoc solid-phase chemistry and formed star-shaped Gd nanoparticles of approximately 240–260 nm after simple in situ mixing of aqueous peptide and GdCl_3_ solutions. Flow cytometry demonstrated that delivery of a fluorescence-labeled phosphopeptide (F′-GpYEEI, 5 µM) increased approximately six-fold in CCRF-CEM cells after 2 h incubation when combined with 50 µM [(WR)_5_C]-GdNPs compared to the phosphopeptide alone. The system also enhanced the chemotherapeutic activity: at 5 µM drug concentration and 50 µM nanoparticle concentration, antiproliferative activity increased by 41% for cisplatin and 18% for carboplatin after 72 h. Drug release experiments using epirubicin showed controlled intracellular release, with about 15% released within 12 h and approximately 60% released by 48 h, demonstrating sustained intracellular drug availability [25].

In another study, the same cyclic peptide–Gd system was further optimized and evaluated specifically as an siRNA delivery platform. Star-shaped nanoparticles (240–260 nm) were formed by mixing 1 mM peptide with 1 mM GdCl_3_. Binding analysis using SYBR Green II showed a strong affinity for siRNA, with a BC_50_ value of 0.044. Zeta potential measurements revealed positive surface charges of +31.7 mV (14 µM) and +36.5 mV (28 µM) for the peptide alone, and +30 mV and +34 mV for the peptide–Gd nanoparticles, enabling efficient complexation with negatively charged siRNA (−15 mV). Cytotoxicity remained low, with approximately 93% cell viability at 50 µM after 48 h in CCRF-CEM and MDA-MB-231 cells. Importantly, fluorescence-activated cell sorting (FACS) showed more than a 10-fold increase in intracellular uptake of Alexa-488-labeled siRNA after 6 h compared to siRNA alone. At an N/P ratio of 40, the nanocomplex reduced STAT-3 protein expression by approximately 62% in MDA-MB-231 cells, confirming effective gene silencing. Together, these studies demonstrate a versatile cyclic peptide–Gd nanoparticle platform capable of enhancing the delivery of both small-molecule drugs and nucleic acids with low toxicity and strong intracellular transport efficiency [24].

Wei et al. [140] developed a multifunctional Gd-doped hollow CeO_2_–ZrO_2_ nanoplatform (Gd/CeO_2_–ZrO_2_/DOX-PEG) for combined chemotherapy and dual-modal MRI/CT imaging by first synthesizing monodisperse Gd-doped CeO_2_ nanospheres (~85 nm) in glycol at 180 °C for 16 h, followed by zirconium incorporation through a Kirkendall effect to generate hollow CeO_2_–ZrO_2_ structures with a high BET surface area of 436.7 m^2^/g and mesoporous architecture (pore sizes 3.8 and 13.2 nm); PEGylation increased the hydrodynamic diameter to 120 ± 10 nm and shifted the surface charge to +16.2 mV, confirming successful surface modification. Doxorubicin (DOX) was loaded into the hollow interior with a loading efficiency of 10.2 wt%, and in vitro release studies demonstrated pronounced pH responsiveness, with ~87% DOX released at pH 6.8 (tumor-like acidic conditions) compared with ~30% at physiological pH 7.4, thereby minimizing premature leakage during circulation. The nanoplatform exhibited a strong dual-imaging performance, with *T*1-weighted MRI phantom studies at 7.0 T showing concentration-dependent signal enhancement (0–0.9 mM Gd) and a longitudinal relaxivity (r_1_) of 4.63 s^−1^mM^−1^, while CT imaging displayed linear attenuation enhancement (0–4.9 mg/mL) with a slope of ~25.23 HU. In vitro assays confirmed the high biocompatibility of empty nanoparticles (>95% HepG-2 viability at 180 µg/mL after 48 h), whereas DOX-loaded nanoparticles induced dose-dependent cytotoxicity, reducing viability to 22.3% at 5 µg/mL DOX after 48 h. In tumor-bearing mice intravenously administered 2.5 mg/kg DOX (seven doses over 22 days), the Gd/CeO_2_–ZrO_2_/DOX-PEG group achieved significantly greater tumor growth inhibition than free DOX without notable body weight loss, and MRI/CT imaging demonstrated peak tumor enhancement at 4 h post-injection, with MRI gray values increasing from 144.3 to 317.2 and CT values from 40.3 to 76.2 HU, confirming effective tumor accumulation and integrated therapeutic–diagnostic functionality within a single nanostructure [140].

Sun et al. (2024) [141] developed bovine serum albumin-coated gadolinium oxide nanoparticles (Gd_2_O_3_@BSA) as a biocompatible nanocarrier for curcumin (CUR) delivery against nasal squamous cell carcinoma, demonstrating nanoscale stability, pH-responsive release, and strong anticancer efficacy. The nanoparticles were synthesized by mixing 0.25 g BSA with 100 mM Gd(NO_3_)_3_ under alkaline conditions (pH 12) with 12 h stirring, followed by 48 h dialysis; STEM imaging revealed spherical, monodispersed particles with core sizes below 10 nm, while dynamic light scattering showed a hydrodynamic diameter of 17 nm that increased to 26 nm after CUR loading, confirming successful drug incorporation. The zeta potential shifted from −32 mV to −36 mV upon CUR loading, indicating enhanced colloidal stability, and UV–Vis spectroscopy confirmed encapsulation through characteristic peaks at ~265–270 nm (Gd_2_O_3_/BSA) and a broad CUR absorption band around 430 nm; the drug loading content reached 21.3%. In vitro release studies demonstrated pH-sensitive behavior, with ~65% CUR released within 10 h at pH 4.0 compared to 43% at pH 7.4, followed by sustained release up to 40 h, supporting preferential drug release in acidic tumor-like environments. Biocompatibility assessments showed excellent safety, with hemolysis below 3% even at 520 µg/mL and nearly 100% viability of normal HFF-2 fibroblasts after 24 h exposure (32.5–520 µg/mL), whereas dose-dependent anticancer activity was observed in CNE-1 and RPMI 2650 carcinoma cells; treatment with Gd_2_O_3_@BSA-CUR (7.5–120 µg/mL) resulted in significantly greater cytotoxicity than free CUR, achieving robust tumor cell growth inhibition at 120 µg/mL, while the blank carrier exhibited minimal toxicity. In vivo evaluation in BALB/c mice administered 25, 50, and 100 mg/kg showed no mortality, no abnormal weight changes, and no histopathological damage in major organs, confirming favorable systemic biocompatibility [141].

Mehdipour et al. presented a computational study of hydrophobic ion pairing using polymyxin B (PMB) and oleate (OA) as a model system to further understand how charged drugs could be transformed to develop more hydrophobic complexes required for drug delivery. Employing dissipative particle dynamics (DPD) simulations with explicit solvents and ions, the authors showed that oppositely charged PMB and OA ions spontaneously interact, forming hydrophobic clusters under a step-growth process. In the first case, electrostatic attraction creates unstable precursors that gradually aggregate into more stable products. This indicates that the charge ratio between PMB and OA is critical for the cluster structure and hydrophobicity and that the peak hydrophobicity is at a 1:1 ratio. Moreover, the composition of the solvent was strongly linked to cluster formation, with higher ethanol content hampering clustering efficiency in ethanol–water mixed systems. The simulations also uncovered two distinct ion-exchange dynamics: the rapid surface exchange of PMB/OA ions and the slower time required to achieve equilibrium between clustered and free ions in solution. The slower relaxation process is of particular interest towards the estimated release of drugs from hydrophobic ion-paired complexes and thus proves important for controlling drug release mechanisms in this way [142].

## 5. GdNPs in Cancer Therapy

Aside from their potential for energy-mediated therapies, GdNPs have been shown to disrupt DNA function and limit cancer cell growth through several mechanisms. GdNPs can inhibit DNA repair pathways, increase oxidative stress, and potentiate radiation-induced double-strand breaks, which cause cell cycle arrest and apoptosis [143,144].

GdNPs have attracted growing interest as anticancer agents in drug delivery systems due to their ability to interfere with DNA function and suppress cancer cell proliferation [74,145]. Owing to Gd’s high atomic number and paramagnetic properties of Gd, GdNPs can amplify radiation-induced DNA damage when used alone or in combination with radiotherapy [118,144]. Upon cellular internalization, these nanoparticles preferentially accumulate in the cytoplasm and nucleus, where they generate ROS either intrinsically or under external stimuli such as X-ray or photon irradiation [118,133]. Excessive ROS levels induce oxidative stress, leading to DNA single- and double-strand breaks, base modifications, and chromosomal instability, which disrupt DNA replication and transcription [146,147]. These molecular events ultimately trigger cell cycle arrest and apoptosis in cancer cells while sparing surrounding healthy tissue when appropriate targeting strategies are employed [131,145].

Beyond indirect DNA damage, GdNPs can be engineered to interact more directly with genetic material or DNA-associated proteins. Surface functionalization by cationic peptides, polymers, or small molecules promotes electrostatic interactions with the negatively charged DNA backbone, facilitating nuclear localization and hindering DNA repair pathways [131]. Multiple studies have shown that GdNPs can antagonize essential DNA repair enzymes including those required for homologous recombination and non-homologous end joining, sensitizing cancer cells to chemotherapeutic drugs and radiation [133,144]. In the context of multifunctional drug delivery platforms, GdNPs are not only active vehicles for cytotoxic drugs or gene-silencing drugs but also serve as adjunctive agents to protect DNA integrity and cancer cell proliferation [147]. The dual characteristics as therapeutic enhancement and intrinsic genotoxic stress render GdNPs as promising materials for next-generation anticancer nanomedicine.

The ability of GdNPs to disturb DNA function and inhibit cancer cell proliferation directly enhances their effectiveness as drug delivery agents through several synergistic mechanisms. One of the most significant contributions is the sensitization of tumor cells to co-delivered therapeutics. GdNPs can promote the generation of ROS, either intrinsically or under external stimulation such as X-ray or photon irradiation, leading to DNA single- and double-strand breaks and replication stress [74,118]. These genotoxic effects commonly induce cell cycle arrest at the G2/M phase, a stage at which cancer cells are particularly sensitive to chemotherapy and radiotherapy [147]. Consequently, drugs delivered in combination with GdNPs exhibit enhanced cytotoxicity, enabling reduced drug dosages while limiting systemic toxicity.

In addition, the DNA-disturbing properties of GdNPs improve intracellular drug retention and nuclear delivery. Activation of DNA damage response pathways results in chromatin remodeling and altered nuclear membrane dynamics, thereby increasing nuclear permeability and the retention of nanoparticle-associated payloads [146]. Surface-engineered GdNPs functionalized with cationic polymers, peptides, or targeting ligands can further enhance electrostatic interactions with the negatively charged DNA backbone, promoting nuclear accumulation of both the nanoparticles and their therapeutic cargo [131]. This property may be a specific asset for DNA-targeting agents such as platinum-based drugs, topoisomerase inhibitors, and gene-regulating therapeutics, as it enhances drug–DNA proximity and overall therapeutic efficacy. Finally, the inherent ability of GdNPs to interfere with DNA repair pathways enables them to act proactively as drug delivery systems rather than passive carriers. GdNPs have been shown to inhibit key DNA repair mechanisms, including homologous recombination and non-homologous end joining, thereby preventing cancer cells from recovering from therapy-induced DNA damage [145,147]. This suppression of DNA repair could reduce the emergence of drug resistance and enhance apoptosis when GdNPs are combined with chemotherapy, radiotherapy, or gene therapy.

GdNPs can enhance drug delivery by co-transporting therapeutic agents, biologically priming cancer cells for amplified therapeutic responses, and simultaneously providing MRI contrast, as demonstrated by Zhang et al., who developed Gd^3+^-doped MgAl layered double hydroxide (Gd-LDH) nanosheets as a combined drug delivery and imaging platform via a co-precipitation method yielding particles with a Mg:Al:Gd molar ratio of approximately 2.1:1:0.05 and an initial hydrodynamic size of 186 ± 1 nm; anticancer drugs were incorporated through ion exchange (400 mg LDH mixed with 390 mg 5-fluorouracil (5FU) or 455 mg methotrexate (MTX) at pH 9.5 for 48 h), increasing particle sizes to 303 ± 4 nm (5FU) and 416 ± 4 nm (MTX), confirming successful loading, with higher drug loading observed for MTX (34.6 ± 5.9 wt%, ~30% encapsulation efficiency) compared to 5FU (13.2 ± 6.2 wt%, ~13% efficiency); structural analyses revealed expanded interlayer spacing and FTIR-confirmed drug incorporation within LDH layers. Drug release exhibited clear pH responsiveness, with MTX release reaching ~80% at pH 5.0, 67% at pH 6.5, and 57% at pH 7.4 after 48 h, while 5FU release reached ~88%, 75%, and 70% at the respective pH values, with ~70% released within the first 2 h via diffusion and ion exchange; stability studies showed negligible Gd release at physiological pH 7.4 and only 1.9–2.9% leaching under acidic conditions after 24 h. Notably, pristine Gd-LDH exhibited high longitudinal relaxivity (r_1_ = 9.5 ± 1.2 mM^−1^s^−1^), approximately double that of the clinical agent Gd(DTPA) (4.5 ± 0.6 mM^−1^s^−1^), and although relaxivity decreased at a neutral pH (~0.83 mM^−1^s^−1^) after drug loading, it increased under acidic conditions to 2.3–2.7 mM^−1^s^−1^ over 24 h, highlighting the system’s high drug loading capacity, controlled pH-sensitive release, physiological stability, and effective MRI contrast performance [50].

## 6. Future Perspectives

A clear near-term direction is the design of ultrasmall, renal-clearable and/or biodegradable GdNPs that preserve high relaxivity while minimizing long-term retention, an especially important translational consideration given the ongoing regulatory and clinical attention to Gd retention and GBCA safety. Recent practice guidance continues to emphasize risk stratification, differences across agent classes, and patient-specific considerations (e.g., renal function), reinforcing the need for next-generation GdNPs with predictable clearance and robust kinetic stability [148].

GdNPs are poised to evolve from “contrast-enabled carriers” into active, therapeutic nanomedicines that integrate delivery, local energy deposition, and real-time treatment monitoring. Beyond MRI, the most impactful expansion of GdNPs is likely in energy-enabled theranostics, where Gd’s high atomic number and magnetic behavior are leveraged to amplify therapy at the disease site while imaging confirms delivery. In oncology, GdNPs are being advanced as radiosensitizers and as multifunctional systems that can pair MRI guidance with radiation enhancement and drug release; hybrid compositions (including high-Z architectures and engineered surface chemistries) are also being explored to improve therapeutic gain under clinically relevant irradiation conditions [116,117]. In parallel, GdNP-enabled neutron capture therapy (Gd-NCT) and cell-assisted delivery concepts (e.g., for glioblastoma targeting) represent a route to overcome rapid clearance and nonspecific biodistribution, which have historically limited Gd-based approaches in these settings [111,118].

From a drug delivery standpoint, future GdNP platforms will likely emphasize programmable biointeractions: (i) stealth-to-adhesive or charge-reversible surfaces that circulate neutrally yet switch to cationic/interactive states within acidic or enzyme-rich microenvironments; (ii) organelle-targeted systems (nucleus/mitochondria/lysosome) to synchronize payload localization with Gd-mediated radiosensitization or ROS stress; and (iii) multi-cargo formulations (drug + immunomodulator + imaging function) to enable treatment sequencing and adaptive dosing. These strategies align with broader trends favoring sub-15 nm constructs for improved tissue penetration and more favorable clearance profiles, while still enabling sufficient payload capacity via high-affinity conjugation chemistries and porous/hybrid architectures [73,93,94].

Clinical translation will ultimately depend on addressing “engineering-to-regulation” gaps: scalable synthesis with tight control of hydrodynamic size distribution, batch-to-batch relaxivity, and leachable/free Gd^3+^; standardized assays for speciation and long-term fate; and harmonized safety packages that address retention, immunotoxicology, reproductive safety, and special populations. The late-stage progress of new, higher-relaxivity macrocyclic agents such as gadoquatrane demonstrates an industry-wide push toward higher imaging efficiency per Gd dose and may indirectly shape the expectations for GdNP products. Future approvals will likely favor platforms that can demonstrate both superior performance and a compelling risk–benefit profile grounded in clearance, stability, and monitoring [113].

## 7. Conclusions

GdNPs were originally developed to be used for MRI imaging through their contrast enhancement capabilities. However, their applications were expanded to be drug delivery systems and theranostics agents. Their special electronic configuration, high atomic number, and flexible coordination chemistry facilitate the simultaneous imaging, radiosensitization, photothermal enhancement, and controlled therapeutic delivery. In contrast to traditional low-molecular-weight Gd chelates, nanoscale formulations have tunable sizes, surface charges, and ligand structures and they feature an optimal control in pharmacokinetics, biodistribution, and tumor targeting. Such properties situate GdNPs as versatile platform materials that have the potential to unify diagnosis and therapy under one construct.

Therapeutically, GdNPs offer synergistic benefits with chemotherapy, radiotherapy and novel energy-based modalities. This ability to amplify radiation-induced DNA damage, produce ROS, and modulate intracellular stress pathways, which would increase anticancer efficacy but reduce the delivery of the cytotoxic agents, through the presence of other targeted cytotoxic agents, is critical due to their potential for potentiation of the pathways. Engineering the surface, such as PEGylation, peptide conjugation and stimulus-responsive coatings, which enhance tumor selectivity and reduce the systemic toxicity, is not only made possible by such surface engineering methodologies as PEGylation, peptide conjugation and stimulus-responsive coatings but also minimizes the tumor-traction. Crucially, the intrinsic MRI visibility of Gd allows for the up-to-the-minute tracking of the nanoparticle biodistribution in the body, a valuable technology in time-sensitive drug planning, response monitoring and treatment response monitoring with personalized tailor-made clinical management of Gd. Despite success in preclinical trials at the preclinical stage, multiple translational hurdles still exist. Long-term Gd retention, speciation, and potential toxicity concerns further highlight the importance of ultrasmall, biodegradable, and kinetically stable formulations with predictable pathways of clearance. Regulatory approval will depend on the systematic assessment of pharmacokinetics, tissue deposition/transformation, and immune compatibility. In addition, scalable and reproducible manufacturing means need to be set up to ensure batch consistency, structural integrity and controlled drug loading at clinically relevant scales. GdNPs represent a compelling frontier in precision nanomedicine. Continued advances in materials chemistry, surface functionalization, and multimodal integration are expected to expand their utility beyond imaging into fully integrated therapeutic systems. With proper attention to safety, biodegradability, and regulatory aspects, GdNP-based platforms hold strong potential for next-generation image-guided drug delivery and personalized theranostics.

## Figures and Tables

**Table 1:** Gd-based photothermal nanoparticles across increasing energy modalities.

Nanoparticle/System	Energy Modality	Major Results	Key Drug Delivery/Theranostic Implications	Reference
Gd_2_O_3_ nanoparticles; Gd-doped hybrid nanostructures	Radiofrequency (RF), NIR light	Efficient non-radiative relaxation → localized heat generation	Hyperthermia	[127]
GdPO_4_/CS/Fe_3_O_4_ scaffold	808 nm NIR laser	Temp ↑ 20 → 47.7 °C in 10 min; tumor apoptosis; BV/TV bone regeneration ↑ to ~61%	Dual-functional platform for tumor ablation + bone regeneration drug delivery	[128]
Microwave-responsive Gd-MOF (Gd/MPC) + anti-PD-1 antibody	Microwave irradiation	Temp ↑ >45 °C in 5 min; tumor inhibition ↑ ~26% vs. free drug	Temperature-triggered immunotherapy drug release	[129]
Gd@Carbon dots loaded with Dox + IR825	808 nm NIR laser	Temp ↑ to ~57 °C; >97% tumor cell kill; strong MRI contrast	Controlled drug release + imaging-guided therapy	[130]

**Table 2:** Representative functionalized GdNPs for enhanced drug delivery, targeting, and controlled release.

GdNP System/Functionalization	Therapeutic Payload	Key Structural Features	Drug Delivery/Biological Outcome	Reference
Chitosan-coated ZnO/TiO_2_/Gd_2_O_3_ nano-composite (RNC)	Rapamycin	Crystalline ZnO, TiO_2_, Gd_2_O_3_; chitosan coating	100% drug loading; sustained release; ROS amplification; apoptosis; G0/G1 arrest in A549 cells	[138]
Solid vs. hollow Gd_2_O_3_ nanospheres with ZnO coating	Doxorubicin	Hollow spheres (~120 nm) with high BET surface area	pH-responsive release; theranostic imaging capability	[139]
Cyclic peptide-GdNPs [(WR)_5_C-Gd]	Small-molecule drugs; siRNA	Star-shaped nanoparticles (240–260 nm); cationic peptide surface	6-fold increased intracellular delivery; enhanced platinum drug efficacy; >10-fold siRNA uptake and STAT-3 knockdown	[25]
Gd-doped hollow CeO_2_–ZrO_2_ nanoplatform (PEGylated)	Doxorubicin	Mesoporous hollow structure; high surface area; PEG coating	pH-responsive release; dual MRI/CT imaging; strong tumor growth inhibition in vivo	[140]
Albumin-coated Gd_2_O_3_ nanoparticles (Gd_2_O_3_@BSA)	Curcumin	Core < 10 nm; hydrodynamic size 17–26 nm; stable colloidal surface	pH-responsive release; high biocompatibility; enhanced cytotoxicity toward nasal carcinoma cells	[141]

## Data Availability

No new data were created or analyzed in this study.

## Abbreviations

The following abbreviations are used in this manuscript:

GdNPS	Gadolinium Nanoparticles
REEs	Rare Earth Elements
ADME	Absorption, Distribution, Metabolism, and Excretion
